# Governing biological material at the intersection of care and research: the use of dried blood spots for biobanking

**DOI:** 10.3325/cmj.2012.53.390

**Published:** 2012-08

**Authors:** Conor M.W. Douglas, Carla G. van El, Alex Faulkner, Martina C. Cornel

**Affiliations:** 1 VU University Medical Center Amsterdam, EMGO Institute for Health and Care Research, Section Community Genetics, Amsterdam, the Netherlands cm.douglas@vumc.nl; 2 King’s College London, Department of Political Economy, London, United Kingdom

## Abstract

A series of governance issues currently surrounds the multiple uses and multiple users of dried blood spots (DBS) for research purposes. Internationally there is a discussion on storing DBS resulting from newborn screening for public health and using them as the basis for large biobank-like collections to facilitate biomedical research. If such a transformation were to be formalized, then DBS would sit at the intersection of care (ie, public health) and research, with the mechanisms through which such a collection could be managed not totally self-evident. What is more, a DBS collection raises questions about the fuzzy boundaries between privacy and anonymity; how to control or define quality control uses of DBS; medical vs nonmedical uses; as well as benefit sharing and stakeholder involvement. Our goal here is to explore some of the key questions relating to DBS governance by way of the bio-objects and bio-objectification concepts. By embracing – rather than resisting to – the blurring of boundaries and problems in categorization that have come to characterize bio-objects and bio-objectification processes recently described in this journal, we attempt to highlight some issues that might not be currently considered, and to point to some possible directions to go (or avoid). Building from our knowledge of the current DBS situation in the Netherlands, we outline questions concerning the uses, management, collection, and storage of DBS.

Establishing and maintaining firm boundaries in biomedical practices are crucially important activities for establishing legal rights and responsibilities, as well as the navigation of routes to regulatory approval of new medicines and products. Classifications delineate what is and is not acceptable within biomedicine, which has knock-on effects in terms of how science, health care, and biomedical research will be structured, organized and funded. However, when such boundaries are breached and classifications begin to breakdown, questions are raised about how biomedicine will be governed.

For instance, an international discussion is currently taking place on storing dried blood spots (DBS) resulting from newborn screening for public health and using them as the basis for large biobank-like collections to facilitate biomedical research (1-3). In some countries, moving these biological materials from the realm of public health screening to that of research has led to public outcry on storage of tissues without proper consent (4-6). As a result processes are under way for considering the reorganization of the procedures for collecting, storing, and using DBS. The goal is to balance public concerns over stored residual material and to provide proper consent *vis-ŕ-vis* the potential of this kind of biobanking initiative (7). Due to the fact that DBS sit at the intersection of care (ie, public health) and research, the mechanisms through which such a collection could be managed are not self-evident. For instance, would these kinds of biological materials be open to any kind of research by any interested party because of their status of residual – or “left-over” – tissue; or would they be subject to scientific and ethical review and on-going informed consent that has to typify biomedical research and development? A formalized DBS collection raises other questions about the fuzzy boundaries between privacy and anonymity – how to control or define quality control uses of DBS; medical vs nonmedical uses; benefit sharing, and stakeholder involvement.

Recently this journal has dedicated space for reflection on how novel biological entities – such as a DBS biobank discussed here – are actively challenging conventional categorizations, classifications, and boundaries in biomedicine. Such entities – or “bio-objects” – have been described as “the products of various efforts to know and enhance (human) life – that is *bio(s) –*, through intervening in and objectifying it, that is through creating often very tangible objects that can be leveraged and stored, as well as circulated and exchanged” (8). Examples of bio-objects are things like frozen gametes that raise questions about the living and non-living categorizations, because they are simultaneously inanimate and the source of vitality (9); microRNA that challenges the boundary between human and non-humans as it migrates from plants to regulate mammalian genes (10), or as in the case presented here DBS biobank that contests the classifications of health research and health care.

In the face of difficulties to classify these biological forms, key governance questions can arise such as: “how to order these entities, who to entrust with their oversight, and in light of what sort of principles?” (11) Our goal here is to explore some of the key questions relating to DBS governance by way of the bio-objects and bio-objectification concepts. By embracing – rather than resisting to – the blurring of boundaries, and problems in categorization, we attempt to highlight some issues that might not be currently considered, and to point to some possible directions to go (or avoid) for the governance of DBS biobanks.

While the article is grounded in our knowledge of the current situation regarding DBS storage, management, and use in the Netherlands that we outline in the next section; many of the questions pertaining to their governance are relevant to similar initiatives taking place around the world. With this overview in place, the core of the article is built around three themed sections that outline questions concerning (a) the uses of DBS, (b) the management of the DBS, and (c) their collection and storage. In our discussion section, we suggest that the governance challenges resulting from the blurring of research and care in DBS biobanking are connected to other boundary issues pertaining to commercial (ie, “private”) and non-commercial (ie, “public”) research, as well as to the use of DBS for quality control of the screening program *vis-ŕ-vis* their use in biomedical research and development.

## Current situation concerning DBS in the Netherlands

In the Netherlands, DBS are collected for neo-natal screening purposes since 1974. Currently, the collection procedure normally takes place as soon as possible after 96 hours of age, and is usually combined with the neonatal hearing screening. A few drops of blood are collected on filter paper and then sent to one of five regional screening laboratories where they are screened for a series of seventeen treatable, developmental disorders like congenital hypothyroidism, metabolic disorders like phenylketonuria, sickle cell disease, and as of May 2011 cystic fibrosis. Since 2002, all Dutch DBS cards are stored for one-year for quality control at one of the regional screening laboratories. With the primary screening purpose of the DBS achieved, the residual tissue is then moved to the Center for Population Screening (CPS) at the National Institute for Public Health and the Environment (RIVM), where they can be used for confirming diagnoses, and sometimes for limited scientific research such as disease prevalence studies. At the moment of screening, parents can indicate if they object to further storage for another four years for research purposes, by ticking an opt-out box on the DBS card itself. Currently in the Netherlands, all cards are destroyed after this total of five years.

While the collection, storage, and use procedure had been stable for the first decades, retention of DBS was publicly questioned in 2000 after a fireworks disaster, when it was suggested that the cards containing the biological samples could be used for forensic identification of the victims. The main points of contention were that it was not well known to parents that DBS cards of their children had been stored, and that no consent had been asked for storage. It was at that time that the five year retention period and consent procedure for research were established.

However, since at least 2005 stakeholders in the Netherlands have been discussing secondary use, geneticists have suggested storing a sample of cards for one or more generations (30 years) to monitor trends in allele frequencies (12). Any changes to the current five-year retention period or to the use of DBS would require action by the Programme Committee Neonatal Heelprick Screening of CPS, which currently advises on policy issues and further development of the screening program. It unites stakeholders that include the patient organizations (VSOP *–* Dutch Parents and Patients Organisation of Genetic Support Groups), medical doctors (eg, metabolic pediatricians), endocrine pediatric experts, obstetricians and clinical geneticists, laboratory officials and members of the Dutch Forum for Biotechnology and Genetics (FBG), a “think-tank” advising the Ministry of Health, Welfare, and Sports. Any request for secondary use is dealt by this Programme Committee Neonatal Heelprick Screening. Until now, requests were mainly made for epidemiological studies and for development of new screening tests. In the literature, suggestions have been made to use the cards for identification and etiological and genome-wide association studies (13). As it has been advised to use existing collections of biological materials in research, setting up a de novo DBS biobank alongside new policies for collection, storage, management, and use could work to avoid legal problems relating to a lack of consent for prolonged storage of the existing DBS samples (14). In the Netherlands, a new law on residual body material (Wet Zeggenschap Lichaamsmateriaal) has been drafted, and meanwhile a code of conduct has been established. A report from the Dutch science and technology policy body – the Rathenau Institute – in 2009 suggested that under certain conditions many people were in favor of doing research on residual material (15). In 2010, Forum FBG contacted the CPS Programme Committee Neonatal Heelprick Screening as to whether prolonged storage would be possible (16). This was discussed with the Ministry of Health, Welfare, and Sport and the CPS was advised to further study the prolonged storage, secondary use, and governance. The Ministry was of the opinion that in potential future scenarios, DBS might best be stored with a non-governmental body or trusted third party (17). As a backdrop to all these discussions, the question remains if the costs and risks to the screening program outweigh the prospective benefits for serving this extension to the research community?

If such a move to prolong the storage and extend the research uses of DBS would be realized, then the public health status of this biological material would begin to blur into the realm of research and development. Such a blurring would not be without its repercussions for the screeners, the organizers of a DBS biobank, and most importantly for parents and the children whose blood is being stored and used. The remainder of this article explores some of the governance questions that arise when considerations are given to this kind of transformative proposal.

## The use of DBS in research

To begin, it is important to differentiate between medical and non-medical uses of DBS. Within the realm of biomedicine, we might try to distinguish between use of DBS for quality control/assurance or for purposes related to the screening program on the one hand, and for research and development on the other. The former use is typically associated with the Center for Population Screening in the RIVM, and the latter is open to actors in the biomedical domain with ethical and scientific approval. As a result it is likely that RIVM wants to keep a firm boundary between the use of cards for research and the use of cards for purposes related to the screening program. This is underlined by the fact that parents do not give permission for, nor are given the option to opt-out of quality control. Therefore, giving consent for screening is giving consent for quality control, as quality control can be seen as an essential part of the screening program. However, it can be debated whether research to optimize the current screening can always be clearly distinguished from research toward new screens? The example of tandem mass spectrometry illustrates how a technology can be used to improve existing tests, and at the same time open up many new possibilities relating to research and extended screening.

While these differentiations in uses stand to be important for the governance of a DBS collection, it may not be advisable to create *a priori* categories for kinds of research that are or are not permissible. This is in part due to fuzzy line between quality control and research, but it is also increasingly difficult to differentiate between fundamental research and its translation into clinical or commercial implications (eg, diseases prevalence or epidemiology studies being translated into research toward development of a new screen).

What is crucial to stress is that certain kinds of research can only – or much more readily and reliably – be done with DBS (see list below). These uses of DBS should be prioritized over other research uses, which may be possible using other research-specific collections.

• Implementation studies of potential new screening techniques (eg, assays for Pompe disease);

• specific clinical epidemiological studies in which DBS from children with specific health problems are studied for potential perinatal causal factors (eg, CMV infections in infants with hearing loss);

• prevalence studies (eg, medium chain acyl coenzyme A dehydrogenase deficiency in newborns).

The priority and acceptability of other purposes still needs to be discussed, eg:

• association studies combining clinical data with DBS (Scandinavian countries);

• identification of victims (eg, Enschede fireworks disaster);

• identification for other forensic purposes.

### Under what conditions could DBS be used for particular kinds of research?

While it is important to keep in mind that biological material containing DNA can never truly be anonymous (18), the use of DBS de-linked from personal health information is less problematic in terms of privacy and public opinion. There are various kinds of linkages between biological information and data that can take various forms. These are summarized in Health Canada Guidance document (19).

It is not self-evident that the future use of DBS for research would be linked to any personal information. This is particularly true given the lack of electronic patient records such as in the Netherlands. It is likely that the information available to link to the DBS would differ during the quality assurance period vs the research retention period. For instance, during the quality assurance period identifiers such as names and contact information must be connected to the DBS for uses such as confirming diagnosis or false positives. In the Netherlands, after this quality assurance period – which currently stands at 1 year – DBS are separated from identifiers.

If requests are made for non-anonymous use it should be shown that samples could not be accessed from other collections; capitalizing on pre-existing biobanks created for research-specific uses should be encouraged. It is the ambition that formalizing the DBS collection would entail the creation of a research biobank that is representative of all newborns. That said, the specific value of a collection representative of all newborns with limited associated data should be weighed against a large but not comprehensive collection with considerable data such as Generation R (ie, a prospective cohort study from fetal life until young adulthood, based in Rotterdam) (20). With that in mind if [further] personal information is desired along with DBS, then permission/informed consent to research participation should be obtained at the time of re-contact for personal information. Nowadays re-contact starts from clinicians or clinical researchers asking parents for consent for a study requiring the DBS of their child. However, this implies linkages between the DBS and – at the very least – personal contact information during the period in which DBS are retained for research. How re-contacting would be practicably done from another way around (ie, the DBS collection to parents) is unclear. It should be stressed that these conditions are likely to be a decision that should be made by a new governance mechanism in the event that the DBS collection is formalized.

### Who could use the cards for research?

In the same way that the specific research uses a DBS biobank, it facilitates biobank’s needs to be in foreground in a discussion on governance. A DBS collection with prolonged retention period would attract researchers interested in effects that go beyond a 5-year cohort. Further, a formalized DBS collection with improved storage facilities including cold storage (eg, minimum -20°C- and preferable -80) would likely serve research related to metabolites (21).

Despite its promise, making decisions about who could use the DBS cards is complicated by the dynamics of contemporary research, which can be conducted by university researchers yet funded –in full or in part – by the commercial sector. Further, contemporary research can also be conducted by public-private-partnerships; and basic research could be done by public institutions, then commercialized by private actors (perhaps via a university industrial liaison office, the creation of a spin-off, etc). One possible resolution to these dynamics and translational mechanisms might be the development of clear benefit sharing agreements (eg, free licensing) resulting from commercialization.

Irrespective of who are the users, the location of research would need to be included in the application to use DBS for research, and made explicit in the material transfer agreement. The dynamics of international research, public-private research, and translational science, mentioned above also apply here as well. Scientific practice is now rarely homogenous and/or contained. This needs to be recognized and integrated into governance mechanisms.

### What are the challenges associated with the use of DBS and genomic technologies?

New technologies such as genome-wide scans, next generation sequencing, and epi-genomics offer new potentialities for the use of DBS in research. These new uses of DBS may pose new questions and raise particular issues. For instance, one-off “informed consent” at time of collection (or even with re-consenting at 18) may be implausible to provide if new technologies open new and unknowable research possibilities. New technologies, such as whole-genome scans, may produce clinically relevant and actionable results. How these results are dealt with, and/or returned to the individual associated with the DBS, needs to be considered. This is something biobanks in general are dealing with, but poses particular challenges in case of a population-based secondary-use biobank, such as in case of the DBS (22). This dynamic of technologies opening research avenues – and concurrent issues – is likely to increase in the future.

## The management and institutions

Currently in the Netherlands, the Programme Committee Neonatal Heelprick Screening is responsible for directing the day-to-day newborn screening activities and handling requests for the use of DBS for research purposes. This Programme Committee is not solely made-up of employees of the screening laboratory and the regional screening organization; it also includes representatives from other relevant stakeholders, as we discussed before. Both Ethical Review Boards and Scientific Advisory Boards are already present in most Dutch University Medical Centers, which would both need to be consulted before a request is made to the Programme Committee for the use of DBS in research.

One institutional mechanism that does not currently exist in the Netherlands is a version of the Community Values Advisory Board (CVAB) that is currently in place in the state of Michigan’s DBS BioTrust (information was provided to us via email by Carrie Langbo – the BioTrust Outreach Coordinator – on March 30, 2012). The CVAB is a board of fifteen to nineteen members selected to reflect geographic diversity and a variety of organizational affiliations and stakeholders that include the general public; cultural, religious or historical groups; health professionals; and disease or health advocacy organizations. The CVAB is one of three boards overseeing the use of DBS for research for the Michigan Department of Community Health (MDCH). The other two are a Scientific Advisory Board and an Institutional Review Board. The CVAB is to play an ongoing role in monitoring the ways in which bloodspots are acquired and used for research; develop a plan for ongoing community awareness, education, and engagement to inform development, review, and revision of BioTrust policies (ie, articulation of recommended formats and vehicles for education and engagement for expectant and new parents, and education and engagement for the community at large); to develop requirements for reports or products that MDCH and/or the BioTrust should provide to the CVAB and to the public at large; as well as mechanisms for the CVAB to provide feedback to MDCH. In particular, emphasis is on advising MDCH on how best to continue educating the community regarding the existence of the BioTrust, use of dried blood spot samples in health research, and individual options for participation or non-participation.

### What role can – or could – parents and other diverse interests play in these institutional mechanisms?

In theory, there would be room for a parent representative on a Scientific Advisory Board, an Institutional Review Board, and an Ethical Review Board. Currently the Dutch Programme Committee Neonatal Heelprick Screening includes patient representatives and in theory there would be room for a parent representative as well. While possible, it is unknown if there currently are parent representatives that sit on these councils or boards – including Community Values Advisory Board in Michigan.

An interesting option for the transparent and socially responsible governance of a DBS collection might be found in an “adaptive governance” model proposed by O’Doherty et al in their work on population-scale biobanks in Canada, where they refer to representation of participants (23). While full description of these mechanisms can be taken from the original it is worthwhile noting that the Participant Association should include all biobank participants who choose to be involved, with funding provided by the biobank to support participant meetings. Members of this Participant Association would select a Participant Board, a group of representatives who are interested in governance and willing to act as a channel between biobank governors and participants. This body of elected representatives would meet as needed and supply members to serve on other biobank committees, such as ethics advisory boards, scientific advisory boards, data access committees, and the Board of Directors (23).

While these institutional mechanisms are directed at bringing donors into the governance process of genomic biobanks, the pediatric nature of the DBS collection would require that the parent-proxies make up the Participant Association and Participant Board.

### How could a governance framework make decisions on the different kinds of research uses?

The inclusion of parents through a mechanism such as a Participants Association or a CVAB would work to broaden the spectrum of voices implicated in the use of DBS for research, still a number of questions remain unanswered. First and foremost, it is not clear who would – or should – decide what kind of governance framework would be in place with a DBS biobank, or who would participate in constructing and carrying out the work of such a framework. Further, if additional bodies were added into a future governance framework, rules would need to be established on how the various boards and committees would work together (ie, would all of the boards have to agree on each use of DBS for research?). In the case of Michigan, the CVAB provided guidance to MDCH in the development of research guidelines for use of DBS in research through the BioTrust. The BioTrust Scientific Advisory Board was implemented by MDCH to convene panels to review and approve each study requesting DBS, and CVAB chose a member of their board to sit on the Scientific Advisory Board. It is policy that this CVAB member is a part of every review panel approving research use of DBS. In whatever formulation that it may take, decisions by a governance framework in the future would need to tackle head-on the dynamics of translational research mentioned above and the associated role of – or need for – benefit sharing agreements.

## The collection and storage of DBS

It is reasonable to think that a new retention period would only apply to new DBS that are collected under the new arrangements. In the Netherlands, existing cards could be used for one plus four years under the pre-existing arrangements, but would need to be destroyed after five years. It is assumed that cards collected under old arrangements should not be used under the new arrangements.

### To what extent – and under what circumstances – would communication and re-contacting be allowed?

Recently, in the Netherlands a lot has been invested in informing future parents about the purpose, collection, and storage of DBS at different moments during pregnancy, and before the actual screening. Yet, more effort can be devoted in informing future parents via midwives and other means, such as Web sites, women’s magazines, public discussions, etc (24). With regards to re-contact, currently parents are only re-contacted if there is a positive result of a screen of the DBS. How therefore re-consent for further information for the use of DBS in research would work in practice (eg, for specific uses of cards for research, or for continued storage at the age of eighteen) is unknown.

### How would informed consent for prolonged storage operate in practice?

Not only could the provision of informational material be adapted to include more on storage, management and use, but training of those discussing the heel prick with the pregnant women (in the Netherlands mostly midwives) need to be optimized and evaluated. In the provision of information on (consenting to) secondary use, the onus should be on safeguarding the original aim of the screening program. In addition, at the time of screening it could be asked whether residual material from the card could be used in research for a certain period, eg, until the child reaches the age of eighteen. This would require a different kind of heel prick card than the one currently used with the opt-out. At the age of eighteen, ethical guidelines agree a person should be able to re-consent (25). Having to answer this question at a moment shortly after birth seems problematic; however, organizing a different moment in time might be too costly.

### Where and how long could/should the DBS be kept for “quality control” and/or research purposes

Currently a card can be used anonymously for quality control of the screening program, for instance for optimizing existing test methods or develop new ones. In the Netherlands, an individual card can be retrieved for quality control, after asking permission from the parents, when a child develops a disorder after screening. It can be ascertained whether the test failed (was false negative) or whether the child acquired the disorder after birth. In case of a false negative further analysis may help reduce future mistakes. In the Netherlands, currently all cards are kept for quality control for one year. However, requests to retrieve the individual card may be made after a few years. Keeping the cards for several years (eg, three to five years) allows for follow-up for a longer period and increases possibilities to compare cases.

The question arises after what period of time other research than quality control could start? The DBS samples should not be too old (depending on the purpose, for enzyme activity until three years, unless kept at -80°C, while DNA can still be retrieved at later stages even when stored at room temperature) and should be stored in appropriate climate conditions, which can be costly. One could already start using the cards for other research purposes after the screening, in case parents have consented. But what would be an optimal moment and format for asking consent (or, less desirable, providing an opt-out)? However, part of the card should be retained for quality control. If there should always (or for a fixed period) be enough material left for quality control purposes, the question is, for how long that could be warranted?

For quality control purposes the cards should be with or within control of the RIVM (currently five screening laboratories). It may not be necessary to actually store them with RIVM, in that case the DBS biobank could have a separate space to store the cards for quality control for a fixed period of time (eg, one to five years), after which cards can be used for other purposes.

## Discussion and conclusion

What we have endeavored to do here is explore some governance questions that relate to biological materials – like a DBS collection – that sits sometimes awkwardly at the intersection of care (ie, public health) and research. We fully recognize that this is not an exhaustive list of issues, yet at the same time similar issues will certainly play a role in other countries and with other bio-objects as well. Attention in the literature is being paid to these matters with important work being done on the regulation of hybrids in tissue engineering and trans-species transplantation (26), and even in biobank governance (27,28). If a decision were made to extend the period of retention for which DBS could be used for research purposes, then the categorization of residual tissue left over from neo-natal screening would increasingly come into flux. Such a transformation of a DBS collection could indeed be read as a kind of bio-objectification process wherein neonates “are first made into objects, [that] become possible, through scientific labor and its associated technologies, and then come to be attributed with specific identities” (29). In the case we explored here one of those specific identities would be that of an institutionalized biobanking research infrastructure. However, as we have seen, such transformative processes do not come without their own sets of complications and challenges on how they are to be justly managed.

It is our view that three interrelated points deserve particular attention in the on-going discussion around DBS governance, which relate to its boundary crossing nature.

The first relates to the blurry lines between commercial and non-commercial research. While we have tried to be clear that a strict distinction between “public” and “private” may be artificial – if not misleading – in contemporary research it is nevertheless a perception that may still be pervasive in public(s) more generally. Delineating and adjusting arrangements between these kinds of research is a real challenge facing the governance of DBS going forward, as is communicating this dynamic to public(s) and DBS stakeholders if and when particular decisions regarding research uses are made.

This problem can be exacerbated by the dynamics of translational medical science, and when the lines between what counts as a quality control use of DBS vs other kinds DBS research uses are unclear. What counts as quality control, who can do quality control, where, when, and for how long need to be clearly articulated in a possible future DBS biobank because the governance arrangements affecting the use of DBS for research will likely differ from the use for quality control.

A the third and final point relates to benefit sharing agreements corresponding to the fuzzy lines between the uses of DBS for quality control vs research by (non)commercial parties. The outlining of different models of benefit sharing is likely to be a worthwhile activity that may help to resolve some of the above-mentioned tensions. If for instance a new screen (developed either through out-right research or quality control work) for a new or existing condition on a newborn screening panel was commercialized (either through a private entity or public-private partnership) then a benefit sharing agreement could allow for its not-for-profit use in a neo-natal screening program but commercial sales elsewhere. Such an arrangement could help to resolve the challenge of differentiating between quality control and research uses of DBS, as well as commercial and non-commercial users. Other models of benefit sharing can be seen in other kinds of biobanks, some including guaranteed access to medicines resulting from biobank-based research or profit sharing from research-related products.

In general, if and when moves are made to set up a de novo DBS biobank it should be clear to parents when the new system starts and what it entails. This process needs time, and it is imperative that such communication is conducted in an understandable, balanced, and transparent manner.

## References

[1] Therrell BL, Hannon WH, Pass KA, Lorey F, Eckman J (1996). Guidelines for the retention, storage, and use of residual dried blood spot samples after newborn screening analysis: Statement of the Council of Regional Networks for Genetic Services.. Biochem Mol Med.

[2] Kharaboyan L, Avard D, Knoppers BM (2004). Storing newborn blood spots: modern controversies.. JLME.

[3] Loeber JG, Burgard B, Cornel MC, Rigter T, Weinreich SS, Rupp K (2012). Newborn screening programmes in Europe; arguments and efforts regarding harmonization. Part 1 – from blood spot to screening result.. J Inherit Metab Dis.

[4] Couzin-Frankel J (2009). Science gold mine, ethical minefield.. Science.

[5] Roser MA (2010). State sued over trading and selling infant blood spots.. New Statesman.

[6] Carmichael M (2011). A spot of trouble.. Nature.

[7] Information on stored newborn screening cards. Available from: http://www.hse.ie/eng/services/healthpromotion/newbornscreening/newbornbloodspotscreening/nscs.html Accessed: August 16, 2012.

[8] Metzler I, Webster A (2011). Bio-objects and their boundaries: governing matters at the intersection of society, politics, and science.. Croat Med J.

[9] Tamminen S. Still life? Frozen gametes, national gene banks and re-configuration of animality. In: Vermeulen N, Tamminen S, Webster A, editors. Bio-objects life if in the 21st century. Farnham (UK): Ashgate; 2012. p.203-18.

[10] Chrupek M, Siipi H, Martinelli L (2012). Bio-objects as “boundary crawlers:” the case of microRNAs.. Croat Med J.

[11] Hansen J, Ingrid Metzler I (2012). Governing bio-objects: a research agenda.. Croat Med J.

[12] Ten Kate LP, Loeber JG, de Knijf P, Bovenberg JA (2005). Trends in our genes. Med Contact (Bussum).

[13] Hollegaard MV, Grauholm J, Borglum A, Nyegaard M, Norgaard-Pedersen B, Orntoft T (2009). Genome-wide scans using archived neonatal dried blood spot.. BMC Genomics.

[14] Koninklijke Nederlandse Akademie van Wetenschappen (KNAW). Multifactorial disorders in the genomics era [in Dutch]. Amsterdam: KNAW; 2006.

[15] Geesink I, Steegers C. Further use further investigated – control of body material [in Dutch]. Den Haag (the Netherlands): Rathenau Instituut; 2009.

[16] Dutch Forum for Biotechnology and Genetics 2010. Letter to the RIVM regarding heel prick card storage. Available from: http://www.forumbg.nl/files/2010-236pdf.doc.pdf Accessed: August 16, 2012.

[17] National Institute for Public Health and the Environment (RIVM). Letter of response to the Forum Biotechnologie & Genetica regarding heel prick card storage 2010. Available from: http://www.forumbg.nl/files/2010-251pdf.pdf Accessed: August 16, 2012.

[18] Wjst M (2010). Caught you: Threats to confidentiality due to the public release of large-scale genetic data sets.. BMC Med Ethics.

[19] Ministry of Health (Canada). Guidance document – definitions for genomic biomarkers, pharmacogenomics, pharmacogenetics, genomic data and sample coding categories. Ottawa: Minister of Public Works and Government Services Canada; 2008.

[20] Generation R. Available from: http://www.generationr.nl/ Accessed: August 16, 2012.

[21] Michopoulos F, Theodoridis G, Smith C, Wilson ID (2011). Metabolite profiles from dried blood spots for metabonomic studies using UPLC combined with orthogonal acceleration ToF-MS: effects of different papers and sample storage stability.. Bioanalysis..

[22] Knoppers BM Deschenes M Zawati MH Tasse AM Population studies: return of research results and incidental findings Policy Statement Eur J Hum Genet 2012Jul 11. [Epub ahead of print] 10.1038/ejhg.2012.152 22781095 PMC3573193

[23] O’Doherty KC, Burgess MM, Edwards K, Gallagher RP, Hawkins AK, Kaye J (2011). From consent to institutions: designing adaptive governance for genomic biobanks.. Soc Sci Med.

[24] Detmar S, Hosli E, Dijkstra N, Nijsingh N, Rijnders M, Verweij M (2007). Information and informed consent for neonatal screening: opinions and preferences of parents.. Birth.

[25] Hens K Van El CE Borry P Cambon-Thomsen A Cornel MC Forzano F Developing a policy for paediatric biobanks: principles for good practice Eur J Hum Genet 2012Jun 20. [Epub ahead of print] 10.1038/ejhg.2012.99 22713814 PMC3533257

[26] Brown N, Faulkner A, Kent J, Michael M (2006). Regulating hybrids: ‘making a mess’ and ‘cleaning up’ in tissue engineering and transpecies transplantation.. Soc Theory Health.

[27] Rial-Sebbag E, Cambon-Thomsen A (2012). Emergence of biobanks in the legal landscape: Towards a new model of governance.. J Law Soc.

[28] Cornel MC Crossing the boundary between research and health care: P3G policy statement on return of results from population studies Eur J Hum Genet 2012Jul 25. [Epub ahead of print] 10.1038/ejhg.2012.160 22828808 PMC3573199

[29] Holmberg T, Schwennesen N, Webster A (2011). Bio-objects and the bio-objectification process.. Croat Med J.

